# Supplementary material for: A preliminary study on assessment of wellbeing among veterinary medical house officers
Source: PLoS One. 2021 Jun 24;16(6):e0253111. doi: 10.1371/journal.pone.0253111 (PMC8224950; doi:10.1371/journal.pone.0253111)
Supplement: Attachment — Submitted filename: Response to Reviewers.docx [file pone.0253111.s001.docx]

**Reviewer #1**:

Thank you for the opportunity to review the article. This topic has become more warranted given more attention is paying for the wellbeing of people who in veterinary and animal welfare profession. I have got a few queries and comments for the authors to consider and address:

*AU: Thank you for taking time to review our manuscript. Below is point-by-point response to your comments. To make it easier for you, we highlighted responses to your comments in green.*

1. The sampled two cohorts of participants in 2017 and 2018. I would like the authors to provide a bit more information (aside from what was written on p. 4, line 92 to 94) on why they recruited in these two different period. Was it because they mainly just want to increase the sample size for analysis?

*AU: Yes, that is correct that we wanted to increase the sample size. This was because data analysis of one of the instruments (Quality of life scores which requires standardization of responses using a special software) for the 2017 cohort was completed in 2018. Therefore, to increase the sample size we enrolled the second group of house officers in 2018.*

*In response to your comment, we added a comment indicating that another reason for enrolling 2 cohorts was to increase sample size. Please see Materials and Methods under “Survey instruments”.*

2. Aside from the authors reporting the Cronbach Alphas for the measures from their own study in the results section, I suggest they also report the measures' initial Cronbach Alphas from the original studies in the Instrument sections.

*AU: We included the Cronbach’s alphas for each instrument. Please see Materials and Methods under “Survey instruments”.*

3. I am a bit confusing with the "Qualitative data collection on perception of level of stress". It looks to me (based on the results) that a scale was used to detect the perception but then there was a free text comments for participants to write qualitative comments in relation to stress. When I read the results, it looks like the authors just reported numbers that matched categories, rather than really engaging with the qualitative data to illustrate the content. This raised the question to me whether the identification of themes provided was mainly based on content analysis. I think the authors need to provide a bit more discussion and distinction. My original thought was that aside from identifying themes from the free texts, there will be some captions be used to further illustrate those themes but that does not seem to be the case. I would probably reframe it as using the score to identify perception of level of stress. Free text enables participants to provide more in-depth descriptions and those descriptions were sorted by content analysis. The way it is being described here would have made other readers to assume some qualitative comments be illustrated to further support the descriptive data.

*AU: Thank you for your comment. Thematic analysis was not performed in this case. And as you indicate, analysis of the free text is simply content analysis. We phrased to indicate that content analysis instead of thematic analysis was performed. Please see Data Analysis and Results (qualitative data collection of perception of levels of stress).*

**Reviewer #2**: 1. Thank you for the opportunity to review this submission.

*AU: Thank you for taking time to review our manuscript. We responded point-by-point to your comments. To make it easier for you to find responses specific to your comments, we highlighted the responses in yellow.*

2. The paper reports on a small-scale cross-sectional survey of well-being among veterinary medical house officers at a training organization in the USA. It would be helpful to have more context, e.g. the responsibilities, support and duration of a veterinary house officer’s role, as not all training programs use this structure.

*AU: Thank you. We added more context regarding the house officers program including responsibilities, duration, and support opportunities. Please see the Introduction.*

3. Data and data analysis
4. Data were collected by a cross-sectional study with 103 respondents, who completed online surveys on anxiety, burnout, depression and quality of life, using validated published scales. The data were collected in 2017 (N=60, 85% female, 34% response rate) and 2018 (43 respondents, 81% female, 36% response rate).
5. Some points to clarify:
6. On p. 16 we learn that it’s a 2-year training program so it is important to clarify whether participants in each year were different cohorts or the same people surveyed twice (a repeated measures design).

*AU: We clarified this in the following ways:*

*1. We included the description of the House Officers Program, including the length of the residency programs and fellowships/internships (Please see the Introduction). Whereas the fellows/interns respondents were different in 2017 and 2018 because they are 1 year-programs, some of residents (unless they were in their final year of the residency program) potentially responded to the surveys in both 2017 and 2018. We did not track the residents who might have responded to the survey in 2017 and 2018 to maintain anonymity of the respondents.*

*2. We also included both 2017 and 2018 in our study to increase our sample size. We indicated this in the Materials and Methods, under the subsection “Survey instruments”. The changes are highlighted in green as Reviewer #1 raised similar concerns.*

7. Participants were asked to rate, not rank, their perceptions of stress associated with the listed stressors, and this is quantitative, not qualitative, data.

*AU: Replaced the word “rank” with “rate” the Abstract and Materials and Methods.*

8. The open-ended questions do provide qualitative data; there is no information on how this was analyzed (e.g. thematic analysis).

*AU: Thank you. Content analysis rather than thematic analysis was performed. We corrected this in the Materials and Methods (Data analysis) and Results section.* *The changes are highlighted in green as Reviewer #1 raised similar concerns.*

9. ‘Selected independent variables’ are mentioned on p. 7 but it’s not clear how they were selected or what they or the dependent/criterion variables were. There is no hypothesis-testing.

*AU: We included the reasons why the chosen independent variables were considered and included relevant references. Please see Data Analysis section.*

*As indicated in our first submission, age was distributed across the various strata considered in our study, but respondents who identified as female and unmarried respondents were overrepresented in our population. We included the data of the multivariate analysis. We discussed the significant variables predicting survey scores, namely gender and marital status. We also discussed age as a variable as it was not a significant predictor of survey scores. We also indicated in the discussion that this should be interpreted with caution due to female and unmarried respondents being overrepresented. Please see results section and discussion. We also included appropriate references in the discussion.*

10. P. 7 states that demographic variables were included; p. 9 states that they were omitted. Without these analyses, there is no information on which groups, if any, were more affected.

*AU: Please see clarification and comments above.*

11. Cronbach’s alpha is given for the scales overall but is also required for each sub-scale, as the analyses were based on these.

*AU: We provided the scales Cronbach’s alpha for all survey instruments in the Materials and Methods, including the subscales for the Maslach Burnout Inventory and Short-Form 8 (Quality of life). We also provided Cronbach’s alpha for the Maslach Burnout Inventory, which has 3 subscales, and Short-Form -8 scale which has 2 subscales, in the results. Please see results. The other survey instruments, namely Generalized Anxiety Disorder, and Patient Health Questionnaire -9 (Depression) only have items without subscales.*

12. There is insufficient information on how the quality of life scores were derived. The burnout measures need number of items and response options.

*AU: Both the Quality of Life (SF-8) and Maslach Burnout Inventory survey instruments are copyrighted. Legally we cannot provide the items. We had access to the instruments through a paid contract. The GAD-7 and PHQ-9 are currently available for use in research and can be accessed online. As indicated in our first submission, we provided as much information as we can without violating any copyrights. We included the number of items for SF-8 and Maslach Burnout Inventory.*

13. The statistical analyses compare scores between the 2017 and 2018 groups. However, there is no testing of sample vs. population distributions.

*AU: We included information of testing distribution in the Data analysis section.*

14. Findings are over-stated. For example, conclusions are made about overall high levels of burnout when scores are on, or within a point of, a threshold and there is wide variation in the data.

*AU: Our interpretation of high burnout was based on higher mean scores of emotional exhaustion and low personal accomplishment scores. As noted in our discussion, other studies indicate that poor perception of personal accomplishment by individual precedes signs of emotional exhaustion. Thus, by the time emotional exhaustion is identified, burnout might be already underway. The respondents in our study had very low scores of perception of their personal accomplishment (<=33).*

*In response to you comment, we revised our results and discussion to make sure our results and their interpretation are not overstated or overreaching. Please see Results and Discussion.*

15. The list of stressors with ‘relatively higher scores’ includes nearly all of them (10 of 13), and 8/13 score 3 on the 5 point scale. As scores are rounded to whole numbers this suggests most were at the scale midpoint, not ‘high’ scores.

*AU: Thank you for the comment. We replaced the phrase “relatively higher scores” with “moderate”. Please see Abstract and Results (qualitative data collection on perception of level of stress).*

16. Overall, the data do not give strong evidence of problems throughout the cohort. More interestingly perhaps, variation in the data suggests that some individuals may be experiencing difficulties, so targeted solutions matched to individual needs may be required.

*AU: As indicated in our introduction, house officers are veterinarians who are potentially under stress during training and our aim was to investigate if their mental wellness is optimal given that veterinary students and other practicing veterinarians have been reported to have elevated psychological stress compared the general population. You are correct, our data does not give strong evidence that that our cohort had higher levels of psychological stress compared to other practicing veterinarians (which was surprising but desirable). We indicated in our discussion proposed ways to offer resources, in this case a faculty member to resolve personal/service concerns. As indicated, one of the major drawback is that our study is preliminary study and has limited external validity, but it might serve as a basis for our/other house officers programs.*

17. Conclusions
18. The paper focuses on problems and argues for interventions but the evidence is thin. There is little said about the null findings for several variables or that, where others exceeded cut-offs, it was never by much. There are considerable problems – logical and statistical – with saying that a score of 49.1 is fine but 50.1 is problematic, for instance.

*AU: Thank you for that comment and your point is well taken. Please see our comment as to why we had focused on “problems” in response to comment #16.*

*Other research recommends focusing on the aspects that improve wellness. In response to your comment, we indicated in the discussion about considering studies that focus on positive aspects, such as measure of happiness. Please see Discussion and relevant references.*

19. As the PHQ is described, it’s not possible to be ‘not at all’ depressed; at best people have to be ‘mildly’ depressed. Depression is a serious diagnosable mental health condition, so telling participants that they are ‘mildly depressed’ based on these scores could cause problems in itself.

*AU: You point is well taken. As indicated in the materials and methods, the PHQ-9 is not a screening tool for depression, but it is validated and used to monitor severity if depression or response to treatment and thus the results in our study should be interpreted as such. The description and use of PHQ-9 were made explicit in our first submission.*

20. Claims that participants ‘consistently experienced high levels of burnout’ do not match the data, from which burnout does not appear to be extreme or widespread.

*AU: Please see our response for item #14 for the response on why we considered our cohort to have high levels of burnout (high emotional exhaustion scores and low scores of personal accomplishment scores). We hope this clarifies the reviewer’s concerns regarding our conclusions on burnout.*

21. The authors suggest that ‘burnout was associated with stressors such as…’ but no tests of association (e.g. correlation) are presented.

*AU: Thank you for the comment. We replaced the word “correlation” with the phrase “might be related to..”*

22. Conclusions about possible intervening variables - coping and resilience, and wider implications e.g. transfer to working environments, are not part of the study.

*AU: We deleted the information regarding wider implications, coping, and resilience. Thank you.*

23. Although there is little evidence of problems, the wider literature is used to argue that there are in fact problems which need to be solved. Scale ranges suggest that some participants may be experiencing problems but this does not show which interventions are required or by whom. Targeted approaches focused on needs would seem preferable. The suggested solutions are not based on the study findings.

*AU: Please see our responses to your comments in #16 and #18 above.*

24. Clarity
25. It’s well written and readable, except that the use of acronyms in the results section means constantly referring back to what each one means. There’s also a constant need to refer back to information on score thresholds to work out what mean scores indicate.

*AU: We responded to the scoring of the content analysis and changed the phrase “relatively higher scores” with “moderate”. Please see responses to comment #15 above.*

*We revised the manuscript and minimized acronyms to reduce distraction to the reader.*

26. Summary
27. Overall, then, this is a small-scale study in one training institution which does not allow conclusions to be drawn about a profession, or even veterinary training, as a whole. The findings are over-stated and focus too much on the negative, and the suggested interventions are unrelated to the study findings. While professional well-being in veterinarians is interesting and important, the conclusions should be carefully aligned with the findings.

*AU: Thank you for the comments. Below is the summarized response for the above comment:*

*1. We revised the manuscript to ensure our conclusions are not overreaching or overstated.*

*2. As pointed out in the introduction, this is a preliminary study and might serve as a basis for future studies.*

*3. We also acknowledged why our study focused on the psychological stress based on previous studies in veterinary students and practicing veterinarians. We also acknowledged that further studies should also focus on aspects of resident training that improve mental health and wellness.*

*4. We also included results of multivariate analysis as you recommended and discussed the results.*

Reviewer #3: Comments for the Authors:

Thank you for the opportunity to review your manuscript. Wellbeing of veterinarians is an important scientific topic and deserves to be investigated.

After reading your manuscript one is confused what the purpose was of carrying out the study in two consecutive school-years? What made you think that veterinarians’ wellbeing may be changed in one year? What happened in that period that might approve this study design? Another question is how many people from the 2017 and 2018 samples are actually the same persons? We don’t know anything about that after reading your description of the results? Further, as the samples from 2017 and 2018 are not dependent nor independent but mixed, there is no statistical test that might compare the respondents wellbeing in 2017 and 2018. In conclusion, you have chosen a wrong method to fulfill the purpose of the study. Therefore, unfortunately I have to recommend the editor to reject your paper.

*AU: Thank you for taking time to review our manuscript. Please see our responses to your comments:*

*1. We included the reason for including 2 cohorts from 2017 and 2018. We included the cohorts to increase our sample size for the cross-sectional study. We indicated this in the Materials and Methods.*

*2. We included the description of the House Officers Program, including the length of the residency programs and fellowships/internships (Please see the Introduction). Whereas the fellows/interns respondents were different in 2017 and 2018 because they are 1 year-programs, some of residents (unless they were in their final year of the residency program) potentially responded to the surveys in both 2017 and 2018. We did not track the residents who might have responded to the survey in 2017 and 2018 to maintain anonymity of the respondents. Anonymity was important because the responses are considered personal medical information.*

*3. We also included multivariate analysis determining variables associated with the survey scores as suggested and discussed the results.*
