# Supplementary material for: A preliminary study on assessment of wellbeing among veterinary medical house officers
Source: PLoS One. 2021 Jun 24;16(6):e0253111. doi: 10.1371/journal.pone.0253111 (PMC8224950; doi:10.1371/journal.pone.0253111)
Supplement: Attachment — Submitted filename: Response to Reviewers.docx [file pone.0253111.s002.docx]

Dr. Wilkinson

Academic Editor

Thank you for reviewing our manuscript. Below are our responses to the reviewers’ comments.

Academic Editor Comments

Thank you for your revisions and responses to reviewers; one of the original reviewers have provide some further comments. I invite you to consider these comments and provide responses, and if necessary, revisions to the manuscript. These comments particular focus on clarity of your work and on ensuring that findings are not overstated.

*AU: We addressed the reviewer’s concerns regarding clarity and conclusions from our study.*

Reviewer #1: Thank you for addressing the comments. I think the manuscript is in good shape to be recommended for publication.

*AU: Thank you.*

Reviewer #2: 1. The focus is on veterinary house officers and the potential stressors they face, which is interesting. It would be helpful to contextualise it with information on other health professionals, but this information doesn't appear until the Discussion. It would be better in the introduction to set the scene for the study. There is good information on the training programme and support available. It would be interesting to know the extent to which house officers use the supports.

*AU: Thank you for your thorough review of our manuscript. Below are our responses to your comments and suggestions. To make it easier for you to identify specific responses to your comments, we highlighted the changes in yellow in the body of the manuscript.*

*We focused on veterinary medicine in the introduction because several studies are available for practicing veterinarians and there are also perceived differences in risk factors for mental health issues among various health professionals (for instance, veterinarians versus physicians).*

2. Some clarification is needed in the method section.

3. Internal consistencies are needed for each scale/subscale based on the data for this study – put this in the Method not the Results. Where reliabilities for the entire measure and for subscales are given, clarify which was used in the analysis and why. For all scales, state how many items were in each subscale, what the scale anchors were, and whether scores were calculated as the sum or mean of items.

*AU: We clarified your comments and suggestions as follows:*

*a. The internal consistency Cronbach alphas we provided in the materials and methods for each instrument (Maslach, GAD-7, PHQ-9 and SF-8) were the original alphas for validation of the instruments (as you requested in the first review). Whereas, the Cronbach’s alphas for the scales/subscales indicated in the results are Cronbach’s alphas from our study. We think it is desirable to indicate the Cronbach’s alphas from our study in the results section so that the reader can relate these to the original alphas reported when the instruments were developed (materials and methods). We had provided these values, but in response to your comment, we clarified the source of the Cronbach’s alphas in the materials and methods (original) and results (from our study).*

*b. We indicated whether scale or subscales Cronbach’s alphas were used in our analysis, and reasons for the choice, namely:*

*Maslach Burnout Inventory (MBI)– we used the 3 subscales emotional exhaustion, depersonalization, and personal accomplishment. The subscales better describe the context of why burnout might have occurred.*

*Generalized Anxiety Disorders (GAD-7) and Patient Health Questionnaire (PHQ-9): The instruments have no subscales therefore whole scale alphas were used.*

*Quality of Life: Subscales mental component summary (MCS) and physical mental summary (PCS) are more relevant than the whole scale because they might guide specific interventions*

*to improve the mental or physical component of quality of life.*

*Please see “data analysis” section for the changes.*

*c. We had indicated the number of items in each instrument in our previous version of the manuscript. Please see materials and methods.*

*d. We had indicated the scale anchors for GAD-7 in our previous manuscript version. We included the anchors for scores 1 (several days) and 2 (more than half the days) for PHQ-9. The anchors for MBI and SF-8 (quality of life) depends on the item, thus, it will be easier for the reader to look at the instrument for specific anchors. As indicated in our previous version of the manuscript, we cannot provide the specific items for the MBI and SF-8 because they are copyrighted. Please see materials and methods for the changes.*

*e. We clarified how the scores were calculated for each instrument. Please note for the MBI we only indicated the number of items used to obtain the scores for the subscales. As indicated in our previous version of the manuscript, a software standardizes the responses against the referent United States population for the SF-8. Please see materials and methods for the changes.*

4. Expand the data analysis section. Which variables met parametric assumptions, which didn’t? Which analysis were carried out using which methods? In some cases medians are given, suggesting non-parametric data, but MANOVA was used. Consistency and clarity in the analysis are required.

*AU: All variables with mean reported followed a normal distribution whereas those with medians reported were not normally distributed.*

*You are correct that a MANOVA (assumes normal distribution) was used and yet some of the data points for some variables were not normally distributed. Because there isn’t a straightforward way in the software we used to run a non-parametric MANOVA, we did the following during the initial data analysis to make sure that running a MANOVA was appropriate:*

*a. We checked for assumptions for using MANOVA to be met by checking whether the residuals were normally distributed and homogeneity of variances was present. MANOVA is fairly robust for underlying distributions and is not sensitive to violations of multivariate normality when the data does not contain outliers.*

*b. To ensure the above in (a), we also ran a MANOVA on ranks and re-analyzed the data and arrived at the same conclusions.*

*We included the clarification summary of relevant information in the data analysis section.*

5. The MBI section states that it ‘gives a context for why burnout could have possibly occurred’ but this is not part of the study – the MBI does not measure the causes of burnout e.g. work demands.

*AU: The “context on why burnout could have possibly occurred” refers to the subscales (emotional exhaustion, depersonalization, personal accomplishment) and not the causes of burnout. Each of the subscales will have different specific reasons for the score magnitude and these have to be investigated. Our statement did not intent to refer to the causes of burnout. We hope this clarifies the reviewer’s comment.*

6. For burnout, avoid calling all subscales ‘burnout’. For instance, p.6: “The emotional exhaustion subscale assesses … indicating low, average and high levels of burnout, respectively” should read “… indicating low, average and high levels of emotional exhaustion, respectively.” Same for depersonalisation and personal accomplishment. This also applies to the Results section e.g. footnote to Table 1.

*AU: We respectfully disagree with the reviewer regarding this comment and interpretation. High emotional exhaustion scores alone, or high levels of depersonalization alone or low scores of personal accomplishment alone are interpreted as indicating burnout. Additionally, a combination of scores for the subscales outside the ranges is also interpreted as indicating burnout. Thus for instance, one cannot conclude a group of individuals who have high emotional exhaustion scores, but within reference depersonalization and personal accomplishment scores are not experiencing burnout. Our argument is based on how the scores are interpreted in the Maslach Burnout Inventory Manual. We did not change the interpretation in the manuscript. We hope the reviewer is comfortable with this comment.*

7. As noted in my earlier review I am concerned that at best a person must be ‘mildly depressed’ on the PHQ-9. How were ethical requirements for avoiding harm managed when giving participants feedback?

*AU: Thank you for the important comment. All identifiers for the respondents were removed and anonymity of respondents was protected before analysis and access by the investigators. The data was reported as median or means for the whole group of house officers. In the event of a house officer requesting individual scores, there were plans to recommend them to discuss the results with Academic and Staff Assistance Program or personal therapist. In our study, none of the house officers requested individual results. It is also important to note that the PHQ-9 is not a screening tool for depression but it is used to monitor severity of depression and response to treatment as noted in the Materials and Methods. Additionally, during administration of instruments, the purpose of each instrument was described. All questions were not delivered in a forced format, and respondents could skip questions.*

8. For the SF-8, how many items were there for the physical and mental scales? What were the scale reliabilities and ranges? How were scores ‘standardised’?

*AU: a. Some items assess both physical and mental components whereas some assess mental component only and others assess the physical component only.*

*For instance the item:*

*a. “Overall, how would you rate your health during the past 4 weeks?” assesses both mental and physical components.*

*Compared to the following items:*

*a. During the past 4 weeks, how much did physical health problems limit usual physical activities (such as walking or climbing stairs)?” assess the physical component.*

*b. During the past 4 weeks, how much have you been bothered by emotional problems (such as feeling anxious, depressed or irritable)?” assesses the mental component summary.*

*We had indicated the scale reliabilities for the mental and physical component summaries. Please also see our response above to why we chose to report the reliability for the mental and physical component summaries. Please see materials and methods under data analysis.*

*As indicated in the materials and methods, responses for each item for each individual respondent are standardized using a software (Optum Pro CoRE). Standardizing refers to converting the responses to scores for the eight dimensions and comparing them to a referent United States population. Please see materials and methods under ‘Quality of life” section.*

9. The rating scales for stressors are quantitative not qualitative as numerical scores are given. The free text answers are, of course, qualitative, but not analyzed here.

*AU: Content analysis (free text) were analyzed as stated in the previous version of the manuscript. Please see under “data analysis” and results section under “quantitative and qualitative data collection on perception of level of stress”*

*We added the word “qualitative” on the subheading “quantitative and qualitative data collection on perception of level of stress” to clarify your comment.*

10. Page 3 states that there were 48 training positions in each year. Two years of data were collected, and p. 9 states that there were 175 potential participants in one year and 119 in the next year. This does not fit the description of the programme.

*AU: The 48 refers to the maximum positions available each year. To clarify, the number of positions available differs each year due the following reasons:*

*a. A program may hire more than one house officer per year whereas some programs may hire a house officer every other year or hire different number each year (for instance 2 house officers in one year and 1 house officer the following year). Thus in a year where all programs are hiring their maximum number of allocated positions approximately 175 participants can be reached; mean of 43 (175/4) house officers per year -1^st^, 2^nd^, 3^rd^ and 4^th^ years in 2017. As indicated the in 2018 only 119 participants were available indicating that some of the house officers graduated and other programs did not hire any house officers or hired less house officers.*

*b. Fellowships and internships are 1 yearlong whereas residency programs are 3-4 years.*

*To clarify we added the word “maximum” before “48” and also added that the number of house officers hired varies depending on the specialty. Please see introduction under information on the residency program.*

11. The demographic information should be summarised in a Table.

*AU: Thank you. We summarized the demographic information in a table (Table 1). We changed the identification of the other tables accordingly. We deleted the sentences with the information on demographic information in the body of the manuscript.*

12. Comparing the two years was not the focus of the study. It would be simpler to state, near the start of the results, that there were no meaningful differences between the two cohorts and to carry out all analyses on the combined datasets.

*AU: While we agree with your comment, we wanted also to demonstrate that the despite the cohorts being dissimilar, the scores were not significantly different. One of the reviewers had also indicated to leave the data as indicated for 2017 and 2018.*

*In response to your comment, we included calculation of combined scores for GAD-7 and PHQ-9, and a column for the combined years form MBI (Table 2) and SF-8 (Table 3). We hope this is acceptable to the reviewer.*

13. Where medians and ranges are given, include the IQR. Alternatively, use mean and SD if suitable.

*AU: Strictly speaking the range is the difference between the minimum and maximum data points and we had provided both the minimum and the maximum data points in the previous version of the manuscript. We do not think it is necessary to provide both the range and IQR as measures of dispersion for the median. In response to your comment, we included IQR as the measure of dispersion for the median because the range is biased and prone to extremely small and large measurements. Please see results.*

*We had provided mean and standard deviation for the measurements that were normally distributed in our previous manuscript.*

14. The findings and Discussion over-state the level of distress identified.

*AU: As we indicated in our previous version, we emphasized the limitations of our study in our discussion mainly focusing on the limited external validity, and overrepresentation of various demographics (biased towards unmarried and or female respondents). Please see also our respondents to your comments regarding interpretation of the survey instruments. We re-read our discussion to make sure our conclusions do not overreach beyond our study results and interpretations.*

15. Anxiety: the medians show ‘mild’ anxiety (p. 6).

*AU: That is correct that our results interpretation of scores of 5-9 are indicative of mild anxiety (Page 6). Median score (for both years) from our study was 5, and that is how we interpreted the results.*

16. Burnout. While emotional exhaustion was high, depersonalisation was average, and feelings of personal accomplishment were on the boundary between low and average. This suggests fatigue/weariness rather than problematic levels of the much more severe syndrome of burnout.

*AU: The scores for personal accomplishment (PA) were 32 (2017), 33 (2018) and 32 (combined). The interpretation for PA scores as indicated in Table 2 are: ≥ 40, 39–34 and ≤ 33 indicate low, average, and high levels of burnout, respectively. The scores are ≤ 33 indicate high levels of burnout. Please see also our responses to #6 regarding interpretation of the Maslach Burnout Inventory. We hope this clarifies the reviewer’s comments.*

17. PHQ-9 ‘mild’ depression - as mentioned above it’s not possible to be better than ‘mildly depressed’. This scale is, by its nature (clinical assessment) designed to show distress. Median scores were around 7 showing, at worst, ’moderate’ levels of depression.

*AU: As mentioned in the materials and methods, the PHQ-9 is not used for screening for depression but it is used to monitor depression or response to treatment. Therefore using the adverb “mildly” implies diagnosis of depression from a screening test/clinical examination. Although this may sound technical, it’s an important differentiation and hence we maintained using the adjective “mild” as recommended by the survey instrument.*

18. No weight can be placed on the gender comparisons as there were so few males; married participants were also under-represented.

*AU: We agree with the reviewer and we had indicated that the results need to be interpreted with caution in the previous version of the manuscript. We retained the comments regarding interpretation of the results regarding gender.*

19. The analysis is unclear. What is meant by ‘when gender [or marital status] was considered as the independent variable, … scores were positively correlated with… ‘? This suggests regression with demographics as control variables (not independent variables) but then the reference to correlation doesn’t fit, and the data analysis section mentions MANOVA – again, not correlation.

*AU:*

*a. To clarify we used the term “explanatory” variable rather than “independent” variable based on your comment.*

*b. “When gender (or marital status) was considered as the independent variables” refers to when either is considered the explanatory variable for the scores. Because of the over-representation of females and unmarried respondents we chose to assess each significant explanatory variable (gender or marital status) one at a time and evaluate its effect on the scores of the 4 instruments. In response to your comment, we simplified, the analysis and re-ran the MANOVA with both gender and marital status in the model. The correlation coefficients were similar and the conclusions remained the same. Please see results.*

*c. It is important to note that each respondent has scores on 4 instruments (GAD-7, PHQ-9, MBI, and SF-8). However, those scores from each respondent on each instrument are not independent, i.e they are correlated. In addition to analyzing variance (similar to ANOVA) between the scores as a function of the explanatory variables (gender and marital status and age in our case) MANOVA also allows investigation of the correlations (strength and direction of the correlation) among the scores from the various instruments while maintaining a single type 1 error (no adjustment for multiple comparisons). The correlations are important information in that they give an idea of the relationship between the scores from the different instruments. For instance, in our study, emotional exhaustion scores were positively correlated with PHQ-9 scores (r=0.59). We hope this clarifies to the reviewer the reason for inclusion of correlations.*

20. The paragraph on p. 12 about the QoL measure is nearly incompressible. As this scale was analysed in terms of two scales – physical and mental – that should be the level of analysis rather than the ‘dimensions’ which are the separate items.

*AU: We understand the reviewer’s comment especially given that we are not able to publish the items, which would make it clear. While the physical and mental component summary are what we used to make interpretations, the scores are derived from the 8 dimensions. We think it is important to include the scores for each dimension as they might help the approaches to interventions when necessary i.e does the intervention need to focus specifically on certain dimensions to improve mental or physical health? For instance, the following item assess the “Social Functioning” dimension: “During the past 4 weeks, how much did your physical health or emotional problems limit your usual social activities with family or friends?” Social functioning is dimension that is affected by either physical, mental or both mental and physical health. Thus, if the score for this dimension is poor, it might be recommended to improve both mental and physical components of quality of life. In contrast the following item assesses physical functioning dimension: “During the past 4 weeks, how much did physical health problems limit your usual physical activities (walking, climbing stairs)?” Intervention for this dimension might be focused on physical component of quality of life.*

*We hope this context is acceptable to the reviewer.*

21. While some scores on the QoL measure were ‘below 50’ they were not far below. A rigid cut-off of 50 provides no information on the variance or standard errors associated with this cut-off. The data show, instead, that the participant group is close to ‘normal’, as would be expected of a hard-working and busy professional group.

*AU: Thank you for the comment. As indicated above the scores from the responses are converted to scores that are compared to a referent American population. In the original validation of the quality of life instrument, the reported standard deviation was 10.*

22. The stress data show that participants endorsed most items, which is to be expected as the items were chosen because they are known sources of stress to working professionals. There’s nothing to say these were problematic stressors for participants.

*AU: We agree with the reviewer’s comments. We only made comments on those items that might be associated with burnout. Furthermore, these items might be only be relevant to our study (for instance physical environment of the teaching hospital) or specific specialty (for instance patient management is irrelevant for an anatomic pathology residency program.*

23. The discussion over-states the levels of distress in this group. Participants appear, by and large, to be doing rather well, although busy and probably at times feeling overwhelmed – that would be normal in a demanding professional programme. Some individuals may be experiencing genuine distress, and may need help and support, but as regards the group, or house officers in general, the data don’t show that they’re overly distressed or unwell. In addition, the study can’t say what’s causing any fatigue/emotional exhaustion – its’ a cross-sectional study and there’s no demonstrated link between the stressor items and the mental health hones. Nothing can’t show causation, the information on work demands is unlinked to the wellbeing data, and some demographic subgroups are too small. The study does not tell us what helps and what doesn’t hep this sample of house officers to manage their wellbeing. Nothing can be said about training as a solution, or that ‘burnout is inevitable’ (p. 18). Much more caution is needed in interpreting the findings.

*AU: Our results focused on burnout for which have evidence of its presence (high emotional exhaustion and low personal accomplish scores). We made a comment that the scores on anxiety and depression were low and we are in fact happy about it (and we gave reasons why are results are different from other practicing veterinarians).* *Burnout results from an individual being overstretched in their work place and if the source of burnout is removed or reduced, the stress experienced by an individual is reduced whereas depression affects one’s holistic life (work and outside work). Burnout can predispose to depression and in our study, it is a concern that need to be addressed.*

*We did not investigate causality but rather associations between explanatory variables and the scores and we were cautious with our interpretations as indicated. The recommendations we indicate in our study are based on other studies in veterinary/medical fields and further studies are required to make such comments. As you point out this is a cross-sectional study, therefore only associations are appropriate.*

*We re-read the discussion to make sure our conclusions are not overreaching.*

Reviewer #3: Thank you again for the opportunity to review the article. You have corrected everything that was required.

*AU: Thank you.*
